# Scope and Prospects of Case Reports in Contemporary Medical Literature

**DOI:** 10.7759/cureus.104643

**Published:** 2026-03-04

**Authors:** Viswanathan Shanthi, Sargunan Arulraja, Paramasivam Anand, Kaliyamurthy Sivaguru, Thangadurai Maheswaran

**Affiliations:** 1 Oral Pathology and Microbiology, Tamil Nadu Government Dental College, Chennai, IND; 2 Pharmacology, Swamy Vivekanandha Medical College Hospital and Research Institute, Tiruchengode, IND; 3 Physiology, RVS Dental College and Hospital, Coimbatore, IND; 4 Prosthodontist and Implantologist, No. 1 Dental Unit Assam Rifles, Shillong, IND; 5 Oral and Maxillofacial Pathology, Adhiparasakthi Dental College and Hospital, Melmaruvathur, IND

**Keywords:** case reports, drug safety, evidence hierarchy, medical literature, rare diseases

## Abstract

Case reports hold an indispensable and enduring position in medical literature and serve as cornerstones for advancing medical knowledge. Despite their placement at the base of the evidence hierarchy, they are unparalleled in their ability to provide early indications of the potential harm of drugs to patients. They illuminate rare and atypical diseases, enrich medical education, and provide a foundation for future studies. With the advent of standardized reporting frameworks and the proliferation of open-access dissemination, the quality and impact of these studies are not only improving but also transforming the medical research landscape. This editorial examines the contemporary relevance of case reports for practicing clinicians and explores their evolution in modern medicine.

## Editorial

Introduction

In today's world, where randomized controlled trials and meta-analyses reign supreme, case reports may seem like mere remnants of descriptive medicine. However, they are far from being outdated. Clinicians frequently encounter cases that challenge existing protocols, such as rare syndromes, unusual drug reactions, and unique comorbidities, for which large-scale studies are insufficient. Case reports are indispensable for bridging these gaps in knowledge. As a unique publication type, they meticulously document individual patient experiences, laying the groundwork for testable hypotheses that can ultimately lead to more comprehensive evidence-based medicine. Their significance has not waned; instead, their roles have evolved and expanded in the modern evidence-based ecosystem of medicine.

Case reports for clinical education

Case reports have long served as cornerstone teaching instruments in the field of medicine. They offer trainees their first encounter with scientific communication and help them develop skills in structured clinical reasoning, literature appraisal and manuscript preparation. Florek and Dellavalle emphasized that case reports commonly represent the initial evidence for new interventions or serve as early alerts that a problem exists with an established therapy, making them equally valuable for training and clinical practice [1]. The iterative process of documenting a case and contextualizing a patient encounter within the existing literature cultivates the habits of intellectual inquiry that distinguish accomplished clinicians from merely competent ones.

Illuminating rare diseases and atypical presentations

In conditions that are too uncommon to recruit adequate trial populations, case reports and case series provide foundational evidence. A comprehensive review of treatable inherited metabolic disorders causing intellectual disability found that 81% of therapeutic evidence was derived from case reports or expert opinions alone, underscoring the indispensability of this format when randomized data are unavailable [2]. Reinforcing this point, Nagtegaal et al. demonstrated that the systematic aggregation of case reports on rare breast cancer metastases to the colorectum yielded prognostic and treatment data comparable to national real-life cohort figures, providing a sound basis for patient counseling and clinical decision-making where no trial evidence exists [3].

Pharmacovigilance and patient safety

One of the most consequential contributions of case reports is pharmacovigilance and the promotion of patient safety. Single careful observations of unexpected adverse drug reactions, off-label therapeutic effects, or rare surgical complications can prompt regulatory scrutiny and guideline revisions long before formal trials are conducted. Riley et al. elaborated on the CAse REport (CARE) guidelines, clearly acknowledging that well-crafted case reports can indicate early signs of possible advantages and risks and provide valuable content for both clinical research and the development of practice guidelines [4]. Without such first-line observations entering the published record, many drug safety signals would remain confined to institutional memory, delaying protective actions for future patients.

Standardized reporting for quality improvement

The criticism that case reports lack methodological rigor is being addressed through the use of structured frameworks. A bibliometric analysis by Trager and Dusek found that adherence to objective, structured reporting formats substantially improves the impact and usability of case reports across research, education, and practice, and that well-documented case reports can directly inform the design of future investigations with higher levels of clinical evidence [5]. The CARE checklist, developed by an international panel and now translated into nine languages and endorsed by numerous peer-reviewed journals, represents the most widely adopted instrument for enhancing completeness and transparency in single-patient case reports [4]. The adoption of such standards is transforming case reports from anecdotal accounts into reproducible and appraisable scientific documents.

Conclusion

Case reports are not merely supplementary to modern medicine; they are indispensable. They play a pivotal role in propelling clinical knowledge beyond the confines of traditional trials, offering invaluable educational insights for future clinicians, and driving pharmacovigilance and hypothesis generation. The implementation of standardized frameworks, such as the CARE guidelines, and the proliferation of open-access journals have significantly elevated the quality and scientific influence of these case reports. It is crucial that clinicians are actively encouraged to meticulously document and publish well-structured case reports. These reports should not be considered a lesser form of scholarship but rather as vital contributions to the ever-evolving landscape of medical evidence. Embracing case reports is not only beneficial but also essential for advancing medical science.
